# Supplementary material for: Acidic Growth Conditions Promote Epithelial-to-Mesenchymal Transition to Select More Aggressive PDAC Cell Phenotypes In Vitro
Source: Cancers (Basel). 2023 Apr 30;15(9):2572. doi: 10.3390/cancers15092572 (PMC10177299; doi:10.3390/cancers15092572)
Supplement: Supplementary file 1 [file cancers-15-02572-s001.zip › Supplementary figures paper Cancers Audero with revisions FINAL.pdf]

Supplementary

Acidic Growth Conditions Promote Epithelial-to-Mesenchymal Transition to Select More Aggressive PDAC Cell Phenotypes In Vitro

Madelaine Magalì Audero, Tiago Miguel Amaral Carvalho, Federico Alessandro Ruffinatti, Thorsten Loeck, Maya Yassine, Giorgia Chinigò, Antoine Folcher, Valerio Farfariello, Samuele Amadori, Chiara Vaghi, Albrecht Schwab, Stephan J. Reshkin, Rosa Angela Cardone, Natalia Prevarskaya and Alessandra Fiorio Pla

Materials and Methods

ATP quantification assay

For ATP quantification assay, cells were plated in 96-well black-bottom polystyrene plates (Greiner Bio-One, Austria) at a density of 3000 cells/well for PANC-1 and 8000 cells/well for Mia PaCa-2 cells in 100 ul medium volume and let adhere overnight in physiological pHe conditions for control, 4 days pHe 6.6 and pHe- selected + 7.4 cell models, while pHe-selected cells were plated in acidic conditions. Acidic cell models were treated with fresh acidic pHe (pHe 6.6) medium the following day, and ATP quantification was assessed at 1h, 48h, and 96h. The number of metabolically active cells was quantified based on the ATP amount using CellTiter-Glo® 2.0 Luminescent Cell Viability Assay (Promega, Cat# G9241) following the manufacturer's indications. Luminescence was recorded using a microplate reader (FilterMax F5, Multi-Mode Microplate Reader, Molecular Devices). Each condition was tested in eight technical replicates, and three independent experiments were performed for each experimental condition.

Single-cell time-lapse cell random migration

PANC-1 cells were plated in technical duplicate in 1% gelatin-coated wells at a density of 5000 cells/well in 500 ul volume in 24-well plates and incubated at 37°C overnight. Before time-lapse imaging, media was changed to remove detached cells. For time-lapse image acquisition, cells were kept at 37°C and under 5% CO_2 in an incubator chamber (Okolab). Cells' random migration was followed in phase contrast illumination at 20X magnification using Eclipse Ti-E Nikon inverted microscope, selecting five representative fields per well, resulting in a total of ten fields per condition and each image stack containing 60 frames. Images were acquired every 10 minutes for 10 hours with a CCD video camera using NIS-Element software (Nikon). Image stacks were analyzed with ImageJ software, and cells were manually tracked using the MtrackJ plugin. Dividing cells and

cells exiting the recorded field were excluded from data analysis, and cells' migratory velocity was used as the parameter. Three independent experiments were performed for each experimental condition.

Immunofluorescence paxillin staining and confocal microscopy

PANC-1 cells seeded on 1% gelatin-coated coverslips were washed twice with cold phosphate-buffered saline (PBS) and fixed with 4% paraformaldehyde for 15 min at 4°C. After washing the samples twice with ice-cold PBS, the fixed cells were then incubated for 10 min at room temperature with PBS containing either 0.1% Triton X-100 (PBST) for permeabilization and blocking of unspecific binding of the antibody was obtained by incubating the cells with PBST containing 1% gelatin and 0.2 M glycine for 30 minutes. The solution was decanted, and a quick wash with cold PBS followed. Cells were incubated in diluted Alexa Fluor® 647 Anti-Paxillin antibody (1:200, ab246719) in PBS containing 1% gelatin overnight at 4°C. The cells were then kept in the dark and washed three times in PBS for 5 minutes each, following DAPI staining for 10 minutes at room temperature. Cells were washed twice with PBS, and coverslips were mounted on glass slides with a drop of Glycergel. Coverslips were sealed with nail polish and stored in the dark at 4°C. Glass slides were then examined using a confocal laser scanning microscope (LSM 700, Carl Zeiss MicroImaging GmbH) with a Plan Apochromat 40x/1.3 numerical aperture oil immersion objective. Peripheral and cytosolic focal Adhesions (FAs) parameters were quantified with ImageJ (NIH, Bethesda, Maryland, USA) by manually selecting at least 10 cells as region of interest (ROI) of 5 representative fields per biological replicate, and paxillin dots were distinguished from the background by applying a threshold, and subsequently, FAs' area and density were automatically quantified by ImageJ software. FAs peripheral translocation was quantified by peripheral FAs to cytoplasmic FAs ratio. The mean numbers obtained from each biological replicate were used for statistical analysis. Three independent experiments were performed for each experimental condition.

Supplementary Figure S1:

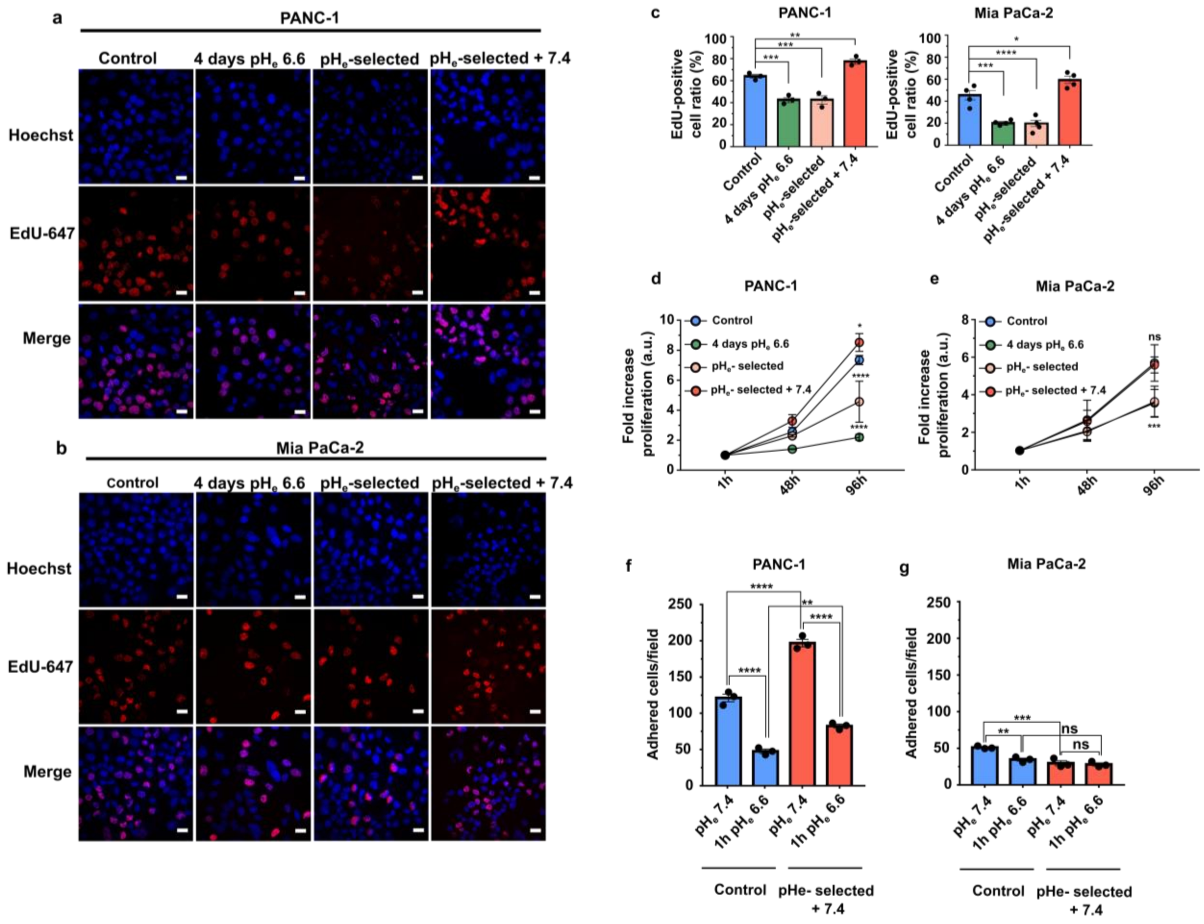

Effect of acidic pHe on cell proliferation, ATP production and of acidic pHe acute treatment of cell-substrate adhesion in PANC-1 and Mia PaCa-2. a) Representative fluorescence images of PANC-1 and **b)** Mia PaCa-2 cell proliferation obtained by EdU staining assay (red, Alexa Fluor 647) and Hoechst (blue) nuclear staining. Scale bar = 20 μm. **c)** Quantification of the percentage of PANC-1 (left) and Mia PaCa-2 EdU-positive cells (right) upon treatment with acidic pHe. Data were reported as the percentage of EdU/Hoechst-positive cell mean ± SEM from 4 representative regions for each condition. **d)** Fold increase in proliferation rate in PANC-1 and **e)** Mia PaCa-2 cells' models assessed by ATP quantification at 1h, 48h, and 96h. Significant differences between PANC-1 or Mia PaCa-2 control vs. all at 96h. **f)** Cell adhesion assay performed on PANC-1 and **g)** Mia PaCa-2 control and pHe-selected cells + 7.4 exposed for 1 hour to pHe 6.6 (acute treatment) before fixation and counting of cells. Data were collected from 4 representative regions for each condition. Each condition was repeated in 8 technical replicates. All data were presented as mean ± SEM from three independent experiments and analyzed using Two-way

ANOVA with Dunnett's multiple comparisons test and One-way ANOVA with Tukey's multiple comparisons test for e) and f), * $p < 0.05$, ** $p < 0.01$, *** $p < 0.001$, **** $p < 0.0001$.

Supplementary Figure S2:

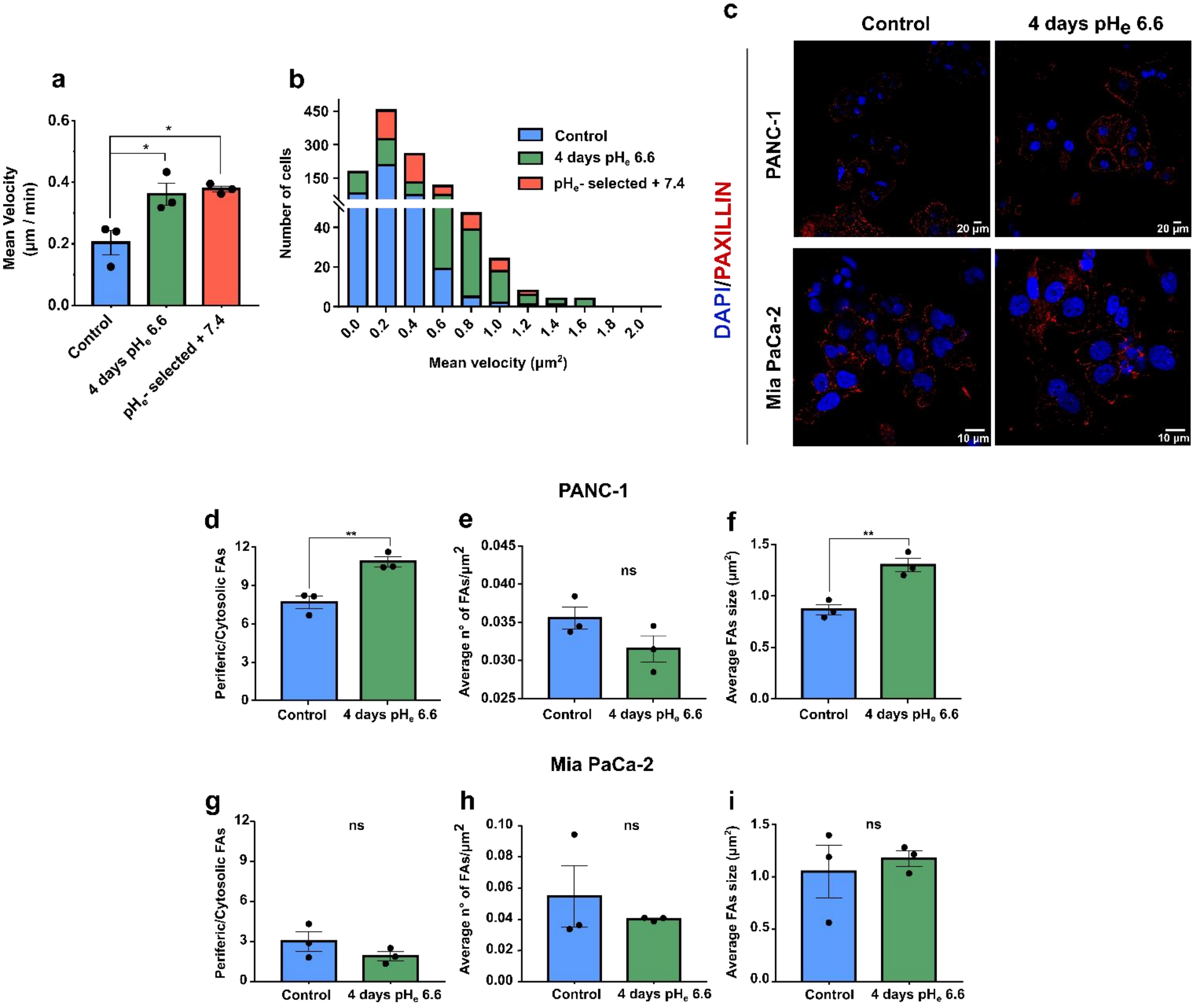

Effect of acidic pH_e on single cell migration in PANC-1 cells and on Focal Adhesions (FAs) membrane recruitment on PANC-1 and Mia PaCa-2 cells. a) Quantification of PANC-1 control, 4 days pH_e 6.6 and pH_e-selected + 7.4 cells' mean velocity (μm/min) obtained by single-cell time-lapse videomicroscopy. Data were collected from five representative regions for each condition (n= 390 cells for control, n= 387 cells for 4 days pH_e 6.6, n= 311 cells for pH_e- selected + 7.4). **b)** Histogram showing the frequency distribution of PANC-1 migration velocities in control cells and in response to 4 days- and 1 month-long (followed by acclimation in 7.4) treatments

in pH_e 6.6 (n= 390 cells for control, n= 387 cells for 4 days pH_e 6.6, n= 311 cells for pH_e- selected + 7.4). **c)** DAPI (blue) nuclei staining and Paxillin (Alexa Fluor 647, red) immunofluorescence staining of PDAC control and 4 days pHe 6.6 cells on gelatin-coated coverslips, obtained by confocal microscopy (Scale bar= 20 µm for PANC-1 cells and 10 µm for Mia PaCa-2 cells). Focal adhesions were observed by staining for paxillin (red). The photos are representative of one field of one experiment. **d and g)** Quantification of the peripheric/cytosolic focal adhesions (FA) ratio, indicative of paxillin recruitment to the focal adhesion, of **e and h)** the average number of focal adhesions (FAs) per μm^2 and of **f and i)** average size (μm^2) of FAs in PANC-1 and Mia PaCa-2 control cells and 4 days pH_e 6.6 cells following paxillin staining (n= 162 cells for control, n= 160 cells for 4 days pH_e 6.6 for PANC-1 cells, n= 88 cells for control, n= 45 cells for 4 days pH_e 6.6 for Mia PaCa-2 cells). All data were reported as mean ± SEM from three independent experiments. Data were analyzed using One-way ANOVA with Dunnett's multiple comparisons test and using an unpaired t-test for d), e), f), g), h), and i), * $p < 0.05$, ** $p < 0.01$, ns not significant.

Supplementary Figure S3:

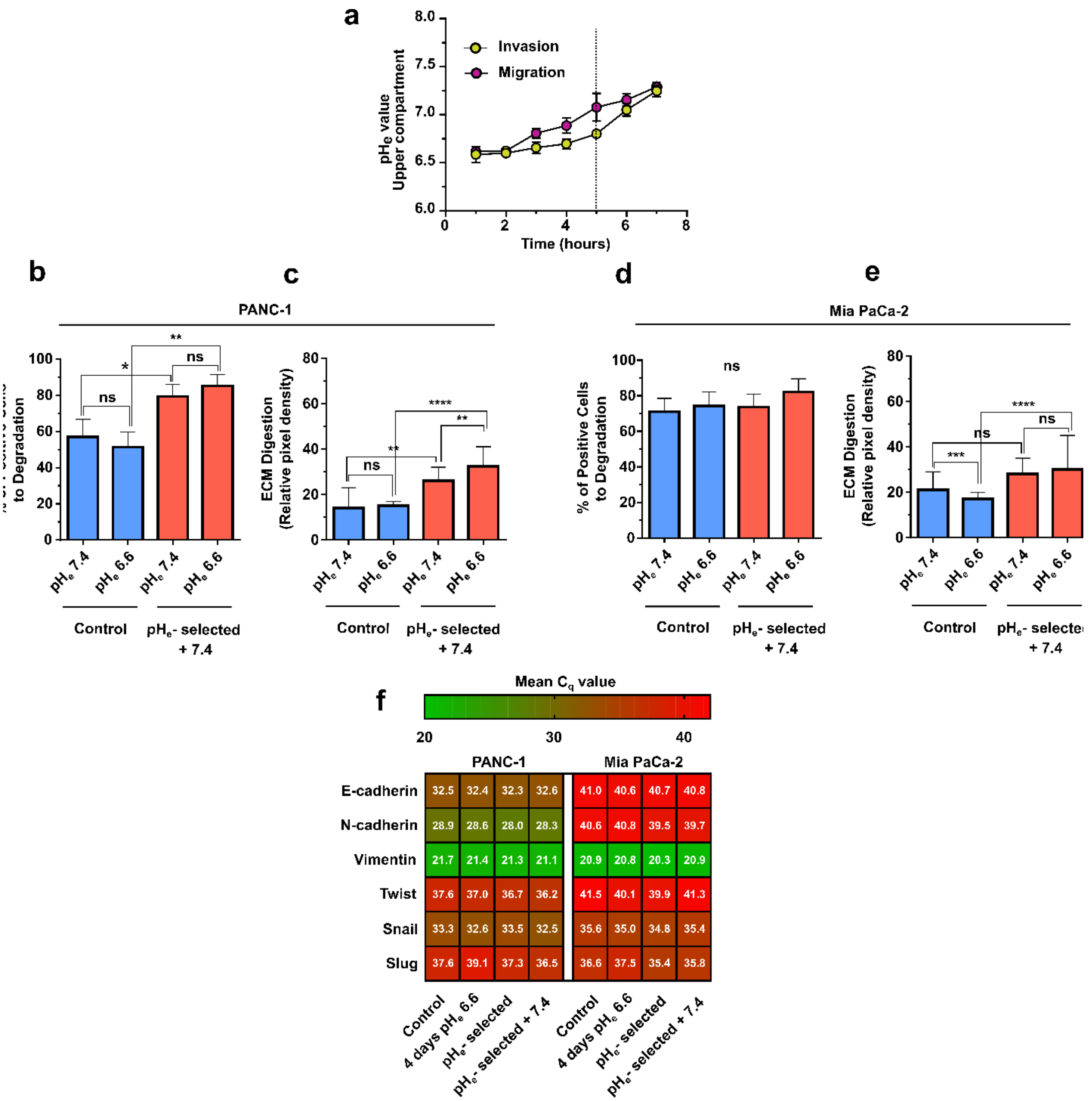

Establishment of a pH gradient in the Transwell system and effect of acute exposition to acidic pH_e on PANC-1 and Mia PaCa-2 cells' invadopodia activity and epithelial-mesenchymal transition. a) Time course indicates the maintenance of pH values of the acidic medium (pH_e 6.6) in the upper compartment of the Transwell system in the presence of pH_e 7.4 in the lower chamber, the presence or absence of a matrigel coating. Cell plates containing the Transwell systems were maintained in the incubator at 37°C and 5% CO_2 to allow pH to equilibrate. Measurements were recorded every hour for 8 hours immediately after removing the plate from the incubator by using pH strips to minimize the time outside the incubator and the alkalinization of the acidic

medium in the upper compartment. **b)** Quantification of the percentage of positive cells to ECM digestion and of **c)** the mean number of invadopodia per cell in PANC-1 control cells and pH_e- selected + 7.4 exposed or not to an overnight exposure to pH_e 6.6. **d)** Quantification of the percentage of positive cells to ECM digestion and of **e)** the mean number of invadopodia per cell in Mia PaCa-2 control cells and pH_e- selected + 7.4 exposed or not to an overnight exposure to pH_e 6.6. **f)** Heatmap showing the RT-PCR mean cycle thresholds (C_q) of PANC-1 and Mia PaCa-2 control, 4 days pH_e 6.6, pH_e- selected, and pH_e- selected + 7.4 cells. Columns represent each condition of the different PDAC cell lines, while rows indicate the mean C_q value of triplicate biological samples, and they are visualized in green and red color scales. All data were reported as mean (± SEM) from three independent experiments, and they were analyzed using Kruskal-Wallis H-test and Dunn's multiple comparison test, * $p < 0.05$, ** $p < 0.01$, ns not significant, *** $p < 0.001$, **** $p < 0.0001$, ns not significant.

Supplementary Figure S4:

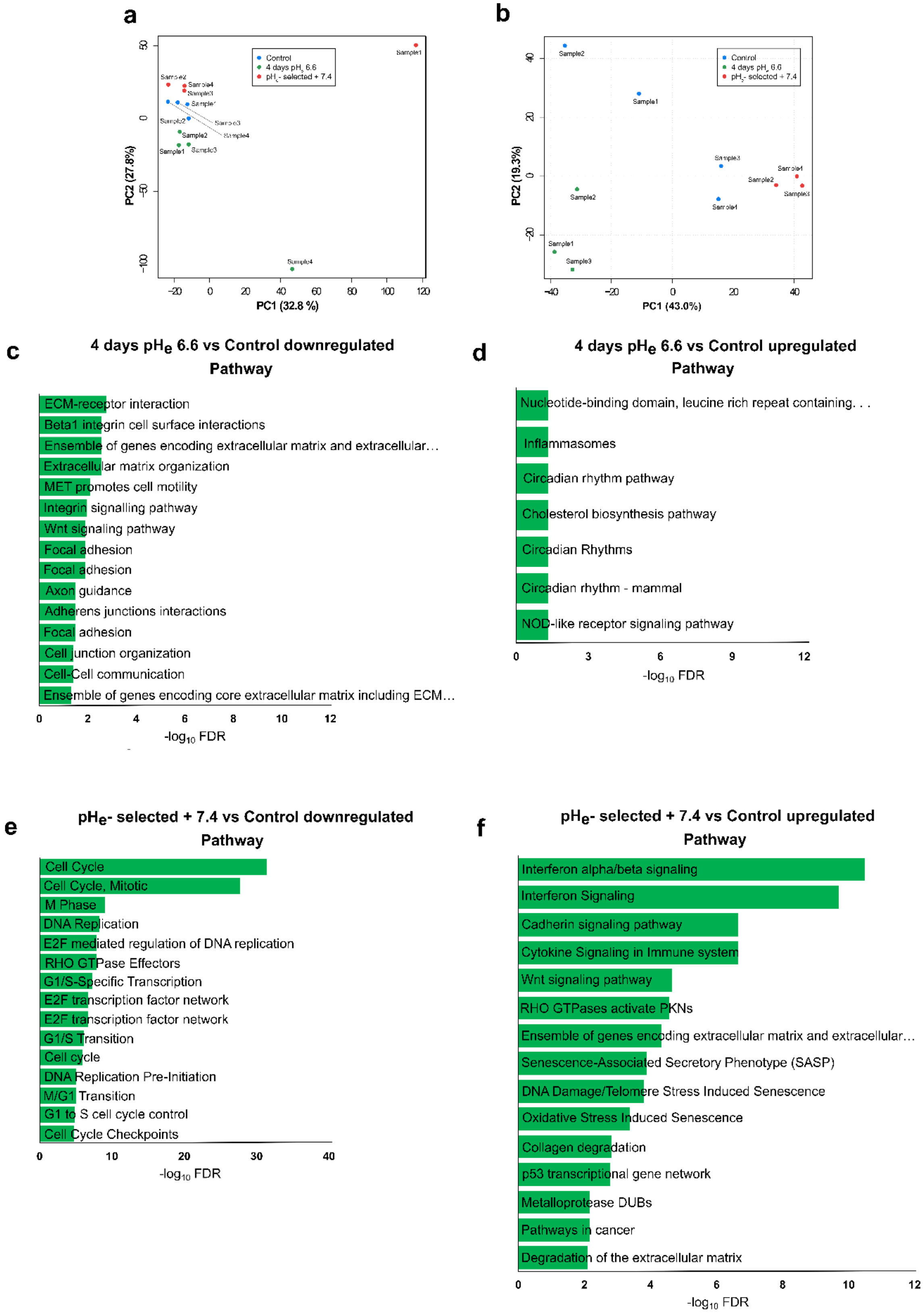

Principal component analysis (PCA) of RNA-seq-based expression data and gene ontology (GO) pathway enrichment analysis of PANC-1 differentially expressed genes (DEGs) in response to acidosis. a) Three-dimensional PCA scatter plots of the first two principal components (PC1, x-axis, PC2, y-axis). PCA was conducted to infer the quality of the RNA-seq data. Each point indicates an RNA-seq sample, and biological replicates are represented with the same colors (red= control, green= 4 days pH_e 6.6, blue= pH_e- selected + 7.4). The processed RNA data formed spatially distinct clusters within the same sample group in the first (PC1) and second (PC2) principal components, indicating similar gene expression profiles. The PCA analysis also revealed differences among RNA-seq data of corresponding conditions (4 days pH_e 6.6 replicate 2 and pH_e- selected + 7.4 replicate 1), mostly due to biological variability of cell populations, therefore the replicates aforementioned were excluded from the samples cohort and **b)** PC analysis was re-performed, showing the segregation of control samples from both acidic conditions. **c)** Bar chart of the most relevant enriched pathway as defined in GO database and resulting from the analysis of genes downregulated in PANC-1 4 days pH_e 6.6 cells vs. Control, and **e)** in PANC-1 pH_e- selected + 7.4 cells vs. Control, and of genes upregulated in **d)** PANC-1 4 days pH_e 6.6 cells vs. Control, and **f)** in PANC-1 pH_e- selected + 7.4 cells vs. Control.
